# Surgical Outcome and Microbial Colonization of Standardized Smear Locations after Pancreatic Head Resection (Pylorus-Preserving Pancreatoduodenectomy, PPPD) for Chronic Pancreatitis and Pancreatic Head Carcinoma

**DOI:** 10.3390/jcm13133810

**Published:** 2024-06-28

**Authors:** Max Grabowski, Ronny Otto, Ina Tammer, Dörthe Jechorek, Henry Ptok, Sara Al-Madhi, Roland Croner, Frank Meyer

**Affiliations:** 1 Department of General, Abdominal, Vascular and Transplant Surgery, Otto-von-Guericke University Medical School with University Hospital, 39120 Magdeburg, Germany; maxgrabowski5@gmail.com (M.G.); sara.al-madhi@med.ovgu.de (S.A.-M.);; 2 Institute of Quality Assurance in Operative Medicine, 39120 Magdeburg, Germany; 3 Synlab Medical Care Center Berlin GmbH, 10829 Berlin, Germany; 4 Institute of Pathology, Otto-von-Guericke University Medical School with University Hospital, 39120 Magdeburg, Germany; 5 Department of General and Abdominal Surgery, Municipal Hospital (“Ernst-von Bergmann-Klinikum”), 14467 Potsdam, Germany

**Keywords:** pancreatic head carcinoma (CA), chronic pancreatitis (CP), pylorus-preserving pancreatic head resection according to Traverso–Longmire, early postoperative outcome, postoperative morbidity, in-hospital mortality, microbial colonization, surgical site infections (SSI), pathogen spectrum, univariate analysis, multivariate analysis

## Abstract

**Introduction:** Patients with chronic pancreatitis (CP) as well as with pancreatic head carcinoma (CA) undergo the surgical intervention named “pylorus-preserving pancreatoduodenectomy according to Traverso–Longmire (PPPD)”, which allowed a comparative analysis of the postoperative courses. The hypothesis was that patients with CA would have worse general as well as immune status than patients with CP due to the severity of the tumor disease and that this would be reflected in the more disadvantageous early postoperative outcome after PPPD. **Methods**: With the aim of eliciting the influence of the different diagnoses, the surgical outcome of all consecutive patients who underwent surgery at the Dept. of General, Abdominal, Vascular and Transplant Surgery at the University Hospital at Magdeburg between 2002 and 2015 (inclusion criterion) was recorded and comparatively evaluated. Early postoperative outcome was characterized by general and specific complication rate indicating morbidity, mortality, and microbial colonization rate, in particular surgical site infection (SSI, according to CDC criteria). In addition, microbiological findings of swabs and cultures from all compartments as well as preoperative and perioperative parameters from patient records were retrospectively documented and used for statistical comparison in this systematic retrospective unicenter observational study (design). **Results**: In total, 192 cases with CA (68.1%) and 90 cases with CP (31.9%) met the inclusion criteria of this study. Surprisingly, there were similar specific complication rates of 45.3% (CA) vs. 45.6% (CP; *p* = 0.97) and in-hospital mortality, which differed only slightly at 3.65% (CA) vs. 3.3% (CP; *p* = 0.591); the overall complication rate tended to be higher for CA at 23.4% vs. 14.4% (CP; *p* = 0.082). Overall, potentially pathogenic germs were detected in 28.9% of all patients in CP compared to 32.8% in CA (*p* = 0.509), and the rate of SSI was 29.7% (CA) and 24.4% (CP; *p* = 0.361). In multivariate analysis, CA was found to be a significant risk factor for the development of SSI (OR: 2.025; *p* = 0.048); the underlying disease had otherwise no significant effect on early postoperative outcome. Significant risk factors in the multivariate analysis were also male sex for SSI and microbial colonization, and intraoperatively transfused red cell packs for mortality, general and specific complications, and surgical revisions. **Conclusions**: Based on these results, a partly significant, partly trending negative influence of the underlying disease CA, compared to CP, on the early postoperative outcome was found, especially with regard to SSI after PPPD. This influence is corroborated by the international literature.

## 1. Introduction

Pancreatic cancer (CA) is one of the most aggressive malignancies, with a 5-year relative survival rate of 10% for both sexes in Germany [1]. Pancreatic cancer is expected to be the second leading cause of cancer death in the US and Germany by 2030 [2,3]. The only treatment option with potential curative intent is radical surgical resection of the tumor lesion by partial pancreaticoduodenectomy (PD), either the classical Kausch–Whipple variant or the pylorus-preserving Traverso–Longmire (PPPD) variant [4]. Corresponding to that in the international literature, the median survival time was reported to be 12–19.2 months for CA after PPPD [5,6] but even longer by recent reports.

Chronic pancreatitis (CP) is a persistent or recurrent inflammation of the pancreas associated with increasing exocrine and endocrine pancreatic insufficiency and pain. In the absence of causal therapy, surgical therapy is recommended in addition to conservative measures, especially in cases of persistent pain or stenosis. In the surgical setting, PPPD is one of the methods used [7].

PPPD is thus used as a surgical procedure for both underlying diseases, with data on in-hospital mortality approximately 5% or less and morbidity ranging from 31 to 58% indicated by results obtained in multicenter studies [8,9,10,11,12,13]. Postoperative infectious complications are of immense importance. These include surgical site infections (SSI), which occur in 35–60% of cases after PPPD [14,15]. The most frequently detected germs in the surgical compartments after PD include *Enterococci*, *Enterobacter* sp., *Klebsiellae*, *Escherichia* (E.) *coli*, *Staphylococci*, and *Candida* sp. [8,16,17,18,19].

The aim of this study was to compare the postoperative surgical outcome in CA and CP having used the same surgical intervention, namely PPPD, with special reference to microbial colonization of various compartments including potential infection as well as SSI.

## 2. Patients and Methods

In the Dept. of General, Abdominal, Vascular and Transplant Surgery of the Otto-von-Guericke University Medical School with University Hospital at Magdeburg, consecutive patients who had undergone PPPD for CP and CA which met the selection criteria were enrolled in this systematic retrospective unicenter observational study (study design: case series); in particular, data were recorded in a computer-based registry over a defined study period.

The descriptive statistical analysis of the medical records was performed by means of a table in Microsoft Excel (2016); this was based on the documentation forms of the “Prospektive Evaluationsstudie elektive Pankreaschirurgie” (Prospective Evaluation Study of Elective Pancreatic Surgery-PEEP, version 1.02) and “PANCALYZE” (version 2.02) [9,20].

During the collection of microbiological data, species of the physiological skin flora were excluded in the absence of clear evidence of pathological relevance.

The primary endpoint was the early postoperative outcome represented by morbidity (in particular, characterized by general and specific complication rate) and in-hospital mortality, especially considering microbial colonization (including surgical site infection [SSI] assessed according to the CDC definition [20]), comparing different underlying pancreatic diseases (inflammation vs. malignancy) with the same surgical procedure, the PPPD (technique, invasiveness, surgical trauma). Specific complications are related to the surgical site field such as bleeding, infections, and fistulas, whereas general complications refer to disorders and problems of other organ systems such as pneumonia, stroke, and myocardial infarction.

Secondary endpoints were possible influencing factors on early postoperative outcome as well as long-term survival of carcinoma patients and quality assurance aspects for reflection of abdominal surgery practice (“real-world data”) at the above-mentioned institution.

Patients with a malignant diagnosis underwent follow-up control examinations, the observation period ended on 1 February 2020. The mean follow-up observation time was 10 years, 7 months and 20 days.

Statistical analysis was performed using IBM SPSS Statistics (version 24; Chicago, IL, USA). Descriptive statistics comprised frequencies, the median, and the interquartile range or the mean and standard deviation. Pearson’s chi-squared test was used to test samples for differences in distribution; for comparison of groups (CA vs. CP), the *t*-test and the Mann and Whitney’s *U* test were used as indicated. *p*-values less than 0.05 were considered significant.

Multivariate analysis was performed by binary logistic or linear regression. Results were described by the regression coefficient, odds ratio (OR), 95% confidence interval (CI), and *p*-value, indicating significant difference. 

The 5-year overall survival rate as well as the mean and median survival times were calculated by the Kaplan–Meier assessment as indicated.

This study was designed and performed anonymously in compliance with the data protection laws of the Federal Republic of Germany and the German District of Saxony-Anhalt.

This study was officially registered under the data file DRKS00023148.

## 3. Results

From 11/2002 to 2015, *n* = 282 records could be fully evaluated according to the study requirements andtherefore entered in the study registry. The underlying diseases were CP in 31.9% (*n* = 90) and CA in 68.1% (*n* = 192). A total of 110 patients were female and 172 were male (sex ratio: m/f = 172:110 [1.56:1]), with a statistically significant difference in gender distribution (*p* = 0.001). The proportion of males was significantly higher in CP than in CA (CP: m/f = 3.1; CA: m/f = 1.2; *p* = 0.001). Comparatively, there was a significant difference in the median age from 68 (CA (range, [37–89]) to 51 (CP [20–82]; *p* < 0.001) years. Patients with CA had a significantly higher median BMI of 25 vs. 23.4 kg/m^2^ (*p* < 0.001) preoperatively, as well as a higher mean ASA score of 2.3 vs. 2.1 than patients with CP (*p* = 0.023). None of the patients received neoadjuvant therapy. The mean duration of surgery was 248.5 (CP) vs. 258.5 min (*p* = 0.068). The number of patients receiving at least one red blood cell pack (RBC) and the mean number of RBCs administered did not differ significantly between the underlying diseases, respectively (*p* = 0.625; *p* = 0.677). Perioperative, i.e., prolonged administration as opposed to “single-shot” antibiotic prophylaxis, was performed in 7.9% in CP vs. 12% in CA (*p* = 0.299). Duration varied from 2 to 10 days, with a median of 7 days. CA patients were significantly more likely to receive application of sandostatin than CP patients (an explanation is the more frequently softer pancreatic parenchyma in CA than in CP patients, with a possibly higher basic risk for anastomotic insufficiency of the pancreaticojejunostomy) and were more likely to undergo additional (partial) vascular resection, according to perceived tumor size and infiltration (*p* = 0.001; *p* = 0.007). Additional (partial) organ resections (nephrectomies as well as atypical partial liver resections) were performed in 18 cases (9.4%) in CA and in 5 cases (5.6%) in CP (*p* = 0.275; Table 1).

There were no significant differences between CA and CP in early postoperative outcome, represented here comparatively by general, specific, and overall complication rates, mortality, and the number of patients with revision. Overall in-hospital mortality was 3.5%. There were 20.6% of all patients affected by at least one general complication and 45.4% by at least one specific complication. The most common specific complication was SSI, which occurred in 28% of all patients. Lymphatic fistulas occurred more frequently in CA with 12% vs. 4.4% in CP (*p* = 0.045). A closer look at the frequencies of each fistula grade A-C in POPF between the two groups revealed that grade C fistulas accounted for 40.9% of all POFP in CA, whereas there were only grade A and B fistulas in CP (*p* = 0.037) (Table 2).

In the univariate analysis of possible factors (other than diagnosis) influencing early postoperative outcome, the following parameters were found to have a significant impact: General complications were more frequent with higher ASA category (*p* = 0.006), increased BMI (*p* = 0.041), longer surgery duration (*p* = 0.016), more intraoperatively transfused RBCs (*p* < 0.01) and additional vascular (partial) resection (*p* = 0.026). Perioperative antibiotic and sandostatin administration (*p* = 0.045; *p* = 0.028, respectively) reduced the incidence of common complications, but the choice of antibiotics used had no effect. With additional partial organ resections and more RBCs transfused intraoperatively, significantly more specific complications (*p* = 0.046; *p* = 0.05) and increased mortality (*p* = 0.01; *p* = 0.02, respectively) occurred postoperatively. The proportion of patients with POPF with applied sandostatin (14.5%) did not differ significantly from those without sandostatin (9.5%; *p* = 0.205). Preoperative biliary drainage (PBD) did not influence general and specific complications and mortality (*p* = 0.586; *p* = 0.338; *p* = 0.696, respectively) (Table 3).

By binary logistic regression, the above-mentioned univariate significant parameters as well as age, gender, BMI, ASA category and the diagnosis (CA vs. CP) were tested for significant influence by multivariate analysis. With increasing intraoperatively transfused RBCs, the general, specific and complication rates in total, the revision rate as well as the mortality were increased (*p* = 0.002; *p* = 0.005; *p* < 0.001; *p* = 0.005; *p* = 0.008, respectively). Also significantly influencing overall complications in terms of increased complication rate multivariately were a higher ASA category and no sandostatin administration. Men also had a 1.825-fold higher risk than women in developing a complication. The more frequent occurrence of lymphatic fistula and POPF grade C in CA compared with CP in univariate analysis was not confirmed in multivariate analysis (lymphatic fistula: *p* = 0.085 (OR (Ref. = CP): 2.980); POPF C: *p* = 0.997).

Diagnosis (CA vs. CP) had no significant impact onto mortality, morbidity, and revisions when tested with multivariate analysis as also not found previously with univariate analysis (Table 4).

### 3.1. Microbiological Results

**Figure 1:**
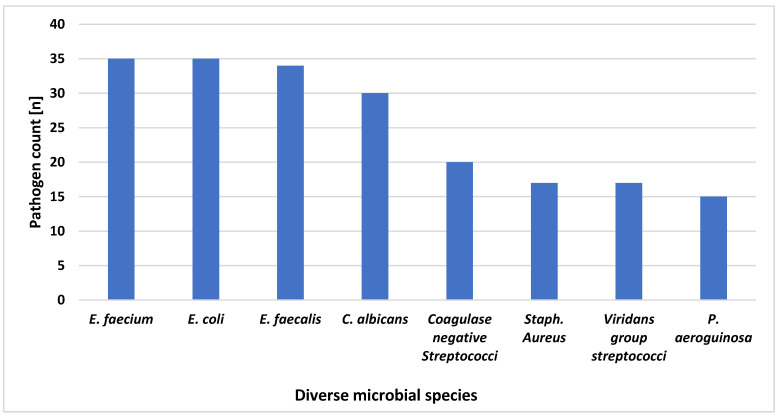
Absolute numbers of the most common pathogens after PPPD.

In total, *n* = 283 relevant pathogens were detected in all operated patients within the time period of 30 days after surgery, *n* = 89 (30.5%) patients were found to be colonized, all surgical compartments as well as urinary tract, respiratory tract, blood and central venous catheters (CVCs) were considered. Of these, *n* = 242 (85.5%) were bacteria and *n* = 41 (14.5%) were fungi. Gram-positive bacteria (47%) were most frequently detected, ahead of Gram-negative (39%) and fungi (14%). The most frequent five individual pathogens detected were, in decreasing order, *Enterococcus faecium* (*n* = 35, absolute), *Escherichia* (*E.*) *coli* (*n* = 35), *Enterococcus faecalis* (*n* = 34), *Candida albicans* (*n* = 30), and *Staphylococcus aureus* (*n* = 17) (Figure 1).

**Figure 2:**
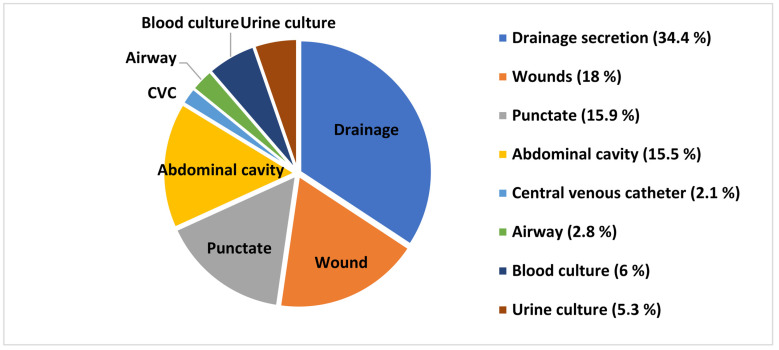
Pathogens detected by smear location, relative to all pathogens determined.

More than three quarters (83.7%) of all pathogens detected were from swabs obtained from surgical field-associated sites, namely drainage secretion (34.3%), wound (18.0%), puncture fluid (15.9%), and abdominal cavity (15.5%); 16.3% of all detections were from swabs obtained from CVCs and airways as well as blood and urine cultures (Figure 2).

Of the 283 germs, relevant resistance was detected in *n* = 14 germs; MRSA, VRE, 3-MRGN and 4-MRGN were considered. This corresponds to a proportion of 4.95%; in all cases, bacteria were detected.

Inclusion of all smear locations as mentioned above showed the following distribution in both patient groups. In CP, 28.9% (*n* = 26) of all patients had proven colonization and 32.8% (*n* = 63) in CA (*p* = 0.509). Comparative analysis of absolute pathogen counts overall as well as of Gram-positive and Gram-negative bacteria and fungi separately showed no significant differences between the two primary diseases. In colonized patients, an average of 3.3 pathogens was detected in CA and 2.9 pathogens in CP (*p* = 0.431).

**Figure 3:**
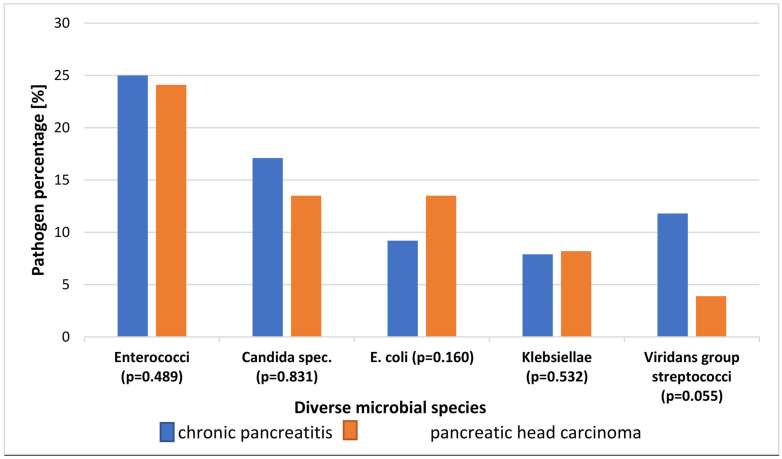
Most common pathogens of underlying diseases, relative to total number of all pathogens in the respective cohort.

Most common pathogens in CA were Enterococci (*n* = 50, absolute), *E. coli* (*n* = 28)/Candida spec. (*n* = 28), and Klebsiellae (*n* = 17); in CP, they were Enterococci (*n* = 19), Candida spec. (*n* = 13), and Viridans group streptococci (*n* = 9). The two patient groups were not significantly different in each type of bug (Figure 3).

When considering surgical site-associated (“SS”) smear locations alone, i.e., wound, abdominal cavity, drainage, and puncture fluid, 24.4% of all patients were confirmed to be colonized with germs in CP and 28.6% of all in CA (*p* = 0.460). In the univariate analysis of other possible pre- and perioperatively influencing factors on the presence of proven postoperative microbial colonization (all smear locations), gender was found to be a significantly influencing parameter: 38.4% of all operated men were colonized, compared to only 20.9% of women (*p* = 0.002). If considering the individual sites with regard to gender-specific differences, a significant difference was found in the “wound” compartment: 10.5% of all men and only 3.6% of all women were colonized there (*p* = 0.037). Gender also had a significant influence on SSI: 33.7% of all men developed it, compared to 19.1% of women (*p* = 0.008).

Preoperative biliary stent placement had no effect on the incidence of colonization or SSI (*p* = 0.815; *p* = 0.414, respectively). However, patients with additional (partial) organ resection were significantly more likely to have SSI than patients without additional resections (47.8% vs. 26.3%; *p* = 0.027). The incidence of POPF correlated significantly with the presence of SSI: SSI occurred in 76.7% of all patients with POPF and 22.2% without POPF (*p* < 0.001). The choice of “single-shot antibiotic prophylaxis” used had no effect on the incidence of SSI. 

Multivariate analysis showed a significant impact of underlying disease on the incidence of SSI: Patients with CA had a 2-fold (OR: 2.025) higher risk than patients with CP of developing SSI (*p* = 0.048). In addition, the influence of gender on the presence of postoperative microbial colonization-as already detected by univariate analysis-was confirmed. The risk for men to be colonized with germs postoperatively was 3.2-fold greater than that for women (OR: 3.244; *p* < 0.001). In addition, men had a 2.3-fold greater risk than women of postoperative SSI (OR: 2.257; *p* = 0.008). 

### 3.2. Long-Term Oncosurgical Outcome of CA Patients

Of the 192 CA patients, 167 (87%) were UICC stage II at the time of surgery, with one case each of cystadenocarcinoma and mucinous non-cystic carcinoma, and the remaining 190 subjects were diagnosed with adenocarcinomas. Nine patients (4.7%) had distant metastasis and more than half had lymph node metastasis (66.7%). An R0 situation could be created surgically in 125 (65.1%).

**Figure 4:**
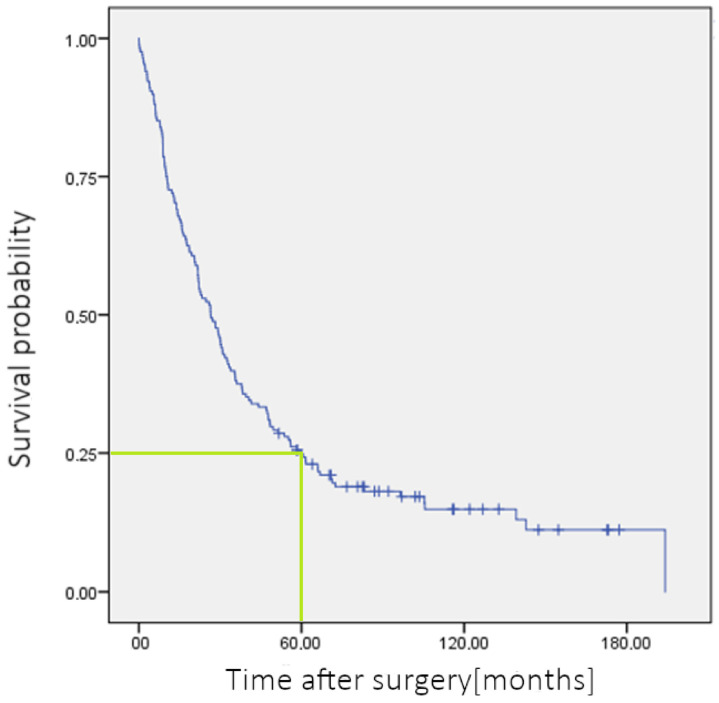
Survival function of CA patients after the PPPD-Kaplan–Meier assessment. Blue color: survival function.

Of the 192 patients operated on, 7 patients died during their stay; of the remaining 185 patients, 161 patients could be followed up and further investigated (87%). On average, survival in the *n* = 168 patients was 50.8 months (mean), with a median of 26.5 months. The 5-year survival rate, calculated by the Kaplan–Meier assessment, was 24.9% (Figure 4).

## 4. Discussion

The present work focused on PPPD, after which—despite the reduction in mortality to less than 5%—high morbidity and associated losses in quality of life still occur in centers with high case volumes [8,9,10,11,12,13]. This warrants further research with the goal of improving the quality of care through better understanding. A role may be played by the possible influence of the underlying disease on the postoperative outcome, and, as characterized here, in particular by microbial colonization and SSI. It was hypothesized that cancer would have a more detrimental effect in general as well as on immune status than chronic pancreatitis and that this would be reflected in alterations of early postoperative outcome.

The in-hospital mortality rate was 3.5%, which was below 5%, comparable to other hospitals designated as “high volume” in the international literature, and well below the 6.0% average for Germany in 2009–2013 proclaimed by Nimptsch et al. [8,9,10,21] Complications occurred in 53.2% of all patients, and the perioperative morbidity is thus in the range of 31–58% according to PPPD as stated in the corresponding literature [11,12,13] but also affected by a very honest and detailed documentation of postoperative abnormalities counted for the calculation of complications and for morbidity. With a revision rate of 9.6%, the above-mentioned hospital showed a value comparable to that in the international literature: in a metastudy, Diener et al. described that reoperations occur in an average of 9.8% of all cases after PPPD [22].

With regard to the SSI rate after PPPD, very heterogeneous values of 6.3%, 35%, and 60% are found in individual publications [14,15,23]. Despite the retrospective study design, the present study succeeded in assessing the presence of SSI uniformly according to the CDC definition and determined an occurrence in 28% of all patients [24].

The metastudies by Chen et al. and Yang et al., and the review by Diener et al., show that there are no significant differences in overall morbidity and wound infections between Kausch–Whipple surgery and PPPD for pancreatic head carcinoma, so that in the case of insufficient studies in PPPD, publications with PD in general were also included but always reported accordingly [12,22,25].

If comparing morbidity after PPPD, there was a non-significant univariate trend towards more general complications in CA compared to CP (23.4% vs. 14.4%; *p* = 0.082), in multivariate analysis patients with CA had a 1.6-fold increased risk (Odds ratio, OR) for general complications, which was also non-significant but reinforced the trend. However, the rates of specific and overall complications were surprisingly close, 45.6% for CA and 45.3% for CP as well as 53.6% for CA and 52.2% for CP, respectively, and did not differ significantly. 

Regarding mortality as a clearly defined hard criterion, the evaluation showed no significant difference with 3.65% in CA versus 3.3% in CP (Table 2). In contrast, in a study of 17,872 PPPD in Germany from 2009 to 2013 published in 2016, Nimpsch et al. described a hospital mortality of 6.3% for pancreatic malignancies compared with 2.0% for chronic pancreatitis [10]. Even though, the category “malignant disease of the pancreas” is not equivalent to pancreatic head carcinoma in all cases, adenocarcinomas represent a majority of all malignant tumor lesions of the pancreas and tumors of the pancreatic head are resected by PPPD, so that due to the high number of cases, contrary to our own observations, a significant difference between CA and CP can be assumed with regard to mortality [4]. This is corroborated by a study from the USA from 1988 to 2003 with 103,222 patients, in which patients underwent various types of pancreatic resections. This showed a 40% higher risk of death during hospitalization with a malignant diagnosis than with a benign diagnosis [26]. Gastinger et al. also showed significantly higher mortality in patients with CA compared with CP in 2003 pancreatic procedures, and Alsfasser et al. described 95% higher hospital mortality for pancreatic cancer compared with chronic pancreatitis in 9566 patients operated on the pancreas in Germany [9,27].

Overall, potentially pathogenic germs were detected in 28.9% of all patients in CP and 32.8% in CA; in the surgical field-associated compartments, the rates were 24.4% in CP and 28.6% in CA; the differences were not significant in either case. The rate of **SSI** was minimally higher in CA (29.7%) than the colonization rate in the surgical field-associated compartments, which can be explained by the fact that microbial detection was not obtained in all cases of SSI. In CP, the SSI rate was equal to the colonization rate (SS) at 24.4. The difference between CA and CP in terms of SSI rate was also not significant, although, as with the colonization rate, it tended to be higher in CA. However, in multivariate analysis, CA diagnosis was shown to be a risk factor for SSI: patients with CA had a 2-fold higher risk of SSI than patients with CP (*p* = 0.048). Regarding microbial colonization rates and SSI, to the best of our knowledge, no direct comparisons between CP and CA after PPPD can be found in the international literature, but single, retrospective unicenter studies comparing benign and malignant diagnosis after PD in general regarding infectious complications: Barreto et al. found no significant differences in postoperative wound infections in 275 subjects after PD in India [28]. The same conclusion was reached by Zhang et al. regarding postoperative SSI, bacteremia, and pneumonia in 212 patients with benign or malignant tumors; in studies by Poruk et al. and Suragul et al. benign and malignant diagnoses were also not predictors of SSI [16,29,30]. Overall, more males than females underwent surgery. The sex ratio was higher in CP with 3.1 (m/f) than in CA with 1.2 (m/f). Although gender had no effect on mortality and overall morbidity, men were found to have a significantly increased risk of SSI (OR: 2.25) and overall microbial colonization (OR: 2.35). Morikane and Okano et al. also described male gender as a risk factor for SSI in studies of 4567 and 4147 PD, respectively, and Farnell et al. showed in 103,222 patients that men had a 30% higher risk of perioperative complications after pancreatic surgery than women [26,31,32]. Only De Pastena et al. concluded that gender had no effect on postoperative infectious complications after 893 PD [33]. Thus, overall, the previous studies, which had a large number of cases and were therefore conclusive, corroborate their own findings. The higher proportion of males in CP might suggest an associated higher risk of SSI in this cohort, but the influence of underlying disease with more SSI in CA seems to counteract this. The number of patients with PBD (preoperative biliary drainage) before surgery did not differ significantly, with a total of 32.2% in CP versus 37.5% in CA. However, when considering the 30-day period before PPPD, significantly more patients with CA (30.2%) than with CP (11.1%) underwent PBD, but there was no significant effect of PDB on overall morbidity and mortality as well as SSI and colonization rate. Gastinger et al. also found no effect of PBD on mortality after PPPD [9]. Sudo et al., Sugiura et al., and Okano et al. also negated the influence of PBD on infectious complications after PD in their publications [8,31,34]. In contrast, three meta-analyses by Gong et al., Chen et al. and Scheufele et al. described PBD as a risk factor for postoperative infectious complications after PD, so that here—contrary to their own results—trends toward more infectious complications after PBD in PD are described in the relevant literature, even if the surgical diagnoses are not limited to CP and CA and PD also includes surgery after Whipple in addition to PPPD [35,36,37].

A modified classification into “high-volume and low-volume surgeon” used by Staufer et al. was also used in this study in an attempt to account for the inclusion of the possible influence of surgeon experience: Although the proportion of operations performed by “high-volume surgeons” was higher in CP (44.4%) than in CA (27.1%), status had no influence on early postoperative outcome [38].

There were no significant differences in intraoperatively administered RBCs between CA and CP. With each RBC administered, the risk for death increased 1.297 fold, for the occurrence of specific and general complications 1.390 fold, and 1.530 fold, respectively, and was thus the strongest factor influencing postoperative outcome in the analysis performed here, but there was no significant influence on SSI and microbial colonization rate. Gastinger et al. also found increased mortality in transfused compared with non-transfused patients after PD [9]. In a study of 4147 PD by Okano et al., significantly more infectious complications occurred after intraoperative administration of RBC, but only tumors and not chronic pancreatitis were operated on there, resulting in poor comparability [31]. 

The choice of antibiotics used after PD, especially with regard to resistance of the local microbial flora, as well as the duration of administration may modulate the risk for SSI [34]. Four different antibiotic combinations were used and documented from 2002 to 2015, and both the choice of antibiotics and their duration of use had no significant effect on SSI and microbial colonization, complications, and mortality in the study shown here. The extent of resections characterized by additional (partial) vessel and/or organ resections, was significantly greater in CA with respect to vessels (9.9% to 1.1%) and organs tended to be greater (9.4% to 5.6%) than in CP, respectively, which seems conclusive in light of the fact that local tumor spread requires greater radicalness with the goal of achieving a R0 resection status. Additional organ resections resulted in more SSI in multivariate analysis.

With an R0 situation in 65.1% of patients operated with pancreatic head carcinoma and a median survival time of 26.5 months with an according 5-year survival rate of 24.9%, the results of the local university hospital compare favorably with international results [5,6].

In principle, the design used here of a retrospective, unicenter observational study with 282 patients seems suitable to compare the early postoperative outcome of two different, defined diseases with the same procedure.

As already shown for mortality and the general complication rate, a (significantly) larger number of cases would have been advantageous to give statistical tests more power. Due to the unicenter and retrospective design, selection bias cannot be avoided, as the study population of a single university hospital cannot be a random selection from the target population, i.e., all patients with CA and CP after PPPD in Germany or worldwide. Multicenter studies could mitigate this effect and, in addition, potentially attenuate existing clinic-specific influential factors. However, a prospective study could improve comparability and external validity by assessing complications according to internationally used definitions, as it is partly performed with DGE in the literature. Furthermore, due the retrospective design, this study had to rely on medical records, which were rigorously checked for containing all relevant information to make statistical testing generalizable. Only three patients had to be excluded due to missing data.

With the results obtained concerning the occurrence of SSI after PPPD as part of the primary outcome, it became possible to assess and partially identify CA as a risk factor. Future studies may build on that data to further reduce complications after PPPD, for example by the approval of different kinds of antimicrobial strategies.

Finally, considering the ongoing discussion on the indicated case volume per year for centers of pancreatic surgery, in particular with regard to pancreatic cancer, the mean number of approximately 30 studies—specifically selected cases per year in the presented study (for both CA and CP but here only in the context of pancreatic head resection)—needs to be further increased step by step to guarantee favorable morbidity and further improve lethality in the near future and the long-term setting.

## 5. Conclusions

Based on the results obtained and presented here, the hypothesis that patients with CA would have a worse general and immune status than patients with CP due to the severity of the tumor disease and the significantly higher age, and that this would be reflected in the early postoperative outcome after PPPD, could not be clearly confirmed. However, there are significant indications for a worse early postoperative outcome in CA after PPPD in the international literature, especially in studies with a large number of cases; and in our own results, there are tendencies and significant indications with regard to SSI. Thus, it can be assumed that chronic inflammation also has a negative influence on the patients’ condition and ultimately on the outcome. In addition, in our results as well as in other publications, other factors such as gender and intraoperative transfusion of RBCs significantly influence the early postoperative outcome and thus possibly reduce or relativize the effects of the underlying disease.

## Figures and Tables

**Table 1:** Comparison of pre- and perioperative baseline data between CP and CA.

	Chronic Pancreatitis	Pancreatic-Head CA	*p*-Value
Total [*n*]	90 (31.9%)	192 (68.1%)	
Age [years] (mean)	52.4	65.6	<0.001 ^(1)^
Gender ratio [*m*/*f*]	3.1	1.2	0.001 ^(2)^
BMI [kg/m^2^] (median)	23.4	25.0	<0.001 ^(1)^
ASA category (mean value)	2.1	2.3	0.023 ^(1)^
Length of stay [d] (mean)	21.1	24.6	0.059 ^(1)^
High-volume surgeon [*n*]	40 (44.4%)	51 (27.1%)	0.004 ^(2)^
Operating time [min] (mean value)	248.5	258.5	0.068 ^(1)^
Patients with at least 1 RBC [*n*]	21 (23.3%)	50 (26%)	0.625 ^(2)^
RBCs administered per patient [*n*]	0.79	0.73	0.677 ^(1)^
Perioperative antibiotics [*n*]	7 (7.9%)	23 (12%)	0.299 ^(2)^
Application of sandostatin [*n*]	13 (14.4%)	63 (32.8%)	0.001 ^(2)^
Patients with additional (partial) vascular resection [*n*]	1 (1.1%)	19 (9.9%)	0.007 ^(2)^
Patients with additional (partial) organ resection [*n*]	5 (5.6%)	18 (9.4%)	0.275 ^(2)^

^(1)^ The *U* test according to Mann and Whitney; ^(2)^ chi-squared test according to Pearson’s test.

**Table 2:** Mortality, morbidity, and revisions comparing CA and CP after PPPD.

	Chronic Pancreatitis	Pancreatic-Head CA	*p*-Value *
Complication (general + specific) [*n*]	47 (52.2%)	103 (53.6%)	0.832
General complication [*n*]	13 (14.4%)	45 (23.4%)	0.082
Specific complication [*n*]	41 (45.6%)	87 (45.3%)	0.970
Revision [*n*]	5 (5.6%)	22 (11.5%)	0.116
In-hospital mortality [*n*]	3 (3.3%)	7 (3.65%)	0.591
SSI	22 (24.4%)	57 (29.7%)	0.361
Number of patients with at least one			
Specific complication [*n*]	41 (45.6%)	87 (45.3%)	0.97
Postoperative pancreatic fistula (POPF)	8 (8.9%)	22 (11.5%)	0.514
Residual pancreatitis	4 (4.4%)	10 (5.2%)	0.783
Fistula of the biliodigestive anastomosis	11 (12.2%)	11 (5.7%)	0.058
Fistula of the gastrointestinal anastomosis	1 (1.1%)	4 (2.1%)	0.564
Delayed gastric emptying	7 (7.8%)	13 (6.8%)	0.759
Ileus	0 (0%)	1 (0.5%)	0.493
SSI	22 (24.4%)	57 (29.7%)	0.361
Intraperitoneal abscess	6 (6.7%)	19 (9.9%)	0.374
Peritonitis	5 (5.6%)	17 (8.9%)	0.336
Burst-abdomen	1 (1.1%)	1 (0.5%)	0.653
Postoperative hemorrhage	6 (6.7%)	17 (8.9%)	0.532
Lymphatic fistula	4 (4.4%)	23 (12%)	0.045

* all chi-squared test.

**Table 3:** Univariate analysis of possible factors influencing complications and mortality, *p*-values.

	General Complication	Specific Complication	In-Hospital Mortality	Test
Diagnosis (CP vs. CA)	0.082	0.97	0.591	(1)
Gender	0.308	0.052	0.166	(1)
ASA category	0.006	0.245	0.125	(2)
BMI	0.041	0.914	0.468	(2)
Age	0.187	0.163	0.485	(2)
Preoperative biliary stent	0.586	0.338	0.696	(1)
Preoperative biliary stent, last 30 d	0.734	0.28	0.757	(1)
High- vs. low-volume surgeon	0.514	0.846	0.857	(1)
Duration of surgery	0.016	0.796	0.089	(2)
Additional (partial) vessel resection	0.026	0.173	0.105	(1)
Additional (partial) organ resection	0.078	0.046	0.01	(1)
Application of sandostatin	0.028	0.685	0.614	(1)
Intraoperatively transfused RBCs	<0.001	0.005	0.002	(2)
Antibiotics used	not significant	not significant	not significant	(2)
Perioperative antibiotic administration	0.045	0.796	0.266	(1)

(1) Pearson’s chi-squared test; (2) binary logistic regression.

**Table 4:** Factors influencing complications and mortality-multivariate analysis using binary logistic regression.

Dependent Variable Independent Variable	*p*-Value	Odds Ratio	95% CI	
			Lower Value	Upper Value
Total complication rate				
Diagnosis (Ref. = CP)	0.302	1.389	0.744	2.594
ASA category	0.281	1.300	0.807	2.094
Intraoperatively (intraop.) transfused RBCs	<0.001	1.660	1.237	2.163
Age	0.182	0.982	0.957	1.008
BMI	0.691	1.013	0.950	1.080
Gender (Ref. = female)	0.020	1.825	1.100	3.027
Specific complication rate				
Diagnosis (Ref. = CP)	0.349	1.349	0.721	2.524
Intraop. transfused RBCs	0.005	1.390	1.106	1.747
Gender (Ref. = female)	0.058	1.679	0.983	2.868
Additional (partial) organ resection	0.148	1.996	0.742	2.954
BMI	0.880	1.005	0.943	1.071
ASA category	0.212	1.353	0.842	2.174
Age	0.100	0.978	0.953	1.004
General complication rate				
Diagnosis (Ref. = CP)	0.229	1.693	0.719	3.989
ASA category	0.013	2.093	1.168	3.751
BMI	0.138	1.063	0.981	1.152
Application of sandostatin (Ref. = no app.)	0.017	0.319	0.125	0.812
Intraop. transfused RBCs	0.002	1.530	1.176	1.988
Perioperative antibiotic administration (Ref. = ot app.)	0.252	0.392	0.079	1.948
Age	0.936	0.999	0.965	1.034
Gender (Ref. = female)	0.682	0.862	0.425	1.750
Duration of surgery	0.176	1.004	0.998	1.010
Additional (partial) vessel resection	0.738	1.233	0.362	4.197
In-hospital mortality				
Diagnosis (Ref. = CP)	0.607	0.633	0.111	3.610
ASA category	0.120	2.620	0.777	8.833
Intraop. transfused RBCs	0.008	1.297	1.070	1.571
Additional (partial) organ resection	0.086	4.178	0.816	21.393
Age	0.420	1.032	0.956	1.114
BMI	0.562	1.050	0.890	1.238
Gender (Ref. = female)	0.473	0.586	0.136	2.521
Revision				
Diagnosis (Ref. = CP)	0.238	2.421	0.557	10.520
Intraop. transfused RBCs	0.005	1.391	1.107	1.748
Gender (Ref. = female)	0.425	1.602	0.503	5.099
BMI	0.998	1.000	0.885	1.129
Age	0.080	0.953	0.903	1.006
ASA category	0.001	4.676	1.883	11.610
Preoperative bilirubin	0.151	1.003	0.999	1.007

## Data Availability

Data is unavailable due to privacy.

## References

[1] Gesellschaft der epidemiologischen Krebsregister in Deutschland (2017). Pankreaskarzinom in Deutschland für 2017/2018.

[2] Rahib L., Smith B.D., Aizenberg R., Rosenzweig A.B., Fleshman J.M., Matrisian L.M. (2014). Projecting Cancer Incidence and Deaths to 2030: The Unexpected Burden of Thyroid, Liver, and Pancreas Cancers in the United States. Cancer Res..

[3] Quante A.S., Ming C., Rottmann M., Engel J., Boeck S., Heinemann V., Westphalen C.B., Strauch K. (2016). Projections of cancer incidence and cancer-related deaths in Germany by 2020 and 2030. Cancer Med..

[4] Seufferlein T., Mayerle J., Benz S., Böck S., Brunner T. (2021). S3-Leitlinie zum exokrinen Pankreaskarzinom. Leitlinienprogr. Onkol. der AWMF DKG DHK.

[5] Seiler C.A., Wagner M., Bachmann T., Redaelli C.A., Schmied B., Uhl W., Friess H., Büchler M.W. (2005). Randomized clinical trial of pylorus-preserving duodenopancreatectomy versus classical Whipple resection—Long term results. Br. J. Surg..

[6] Tran K.T.C., Smeenk H.G., van Eijck C.H.J., Kazemier G., Hop W.C., Greve J.W.G., Terpstra O.T., Zijlstra J.A., Klinkert P., Jeekel H. (2004). Pylorus Preserving Pancreaticoduodenectomy Versus Standard Whipple Procedure. Ann. Surg..

[7] Beyer G., Hoffmeister A., Michl P., Gress T.M., Huber W., Algül H., Neesse A., Meining A., Seufferlein T.W., Rosendahl J. (2022). S3-Leitlinie Pankreatitis—Leitlinie der Deutschen Gesellschaft für Gastroenterologie, Verdauungs- und Stoffwechselkrankheiten (DGVS) 1—September 2021—AWMF Registernummer 021-003. Z. Gastroenterol..

[8] Sugiura T., Uesaka K., Ohmagari N., Kanemoto H., Mizuno T. (2012). Risk Factor of Surgical Site Infection after Pancreaticoduodenectomy. World J. Surg..

[9] Gastinger I., Meyer F., Shardin A., Ptok H., Lippert H., Dralle H. (2019). Untersuchungen zur Hospitalletalität in der Pankreaschirurgie. Der. Chirurg..

[10] Nimptsch U., Krautz C., Weber G.F., Mansky T., Grutzmann R. (2016). Nationwide In-hospital Mortality Following Pancreatic Surgery in Germany is Higher than Anticipated. Ann. Surg..

[11] Taher M.A., Khan Z.R., Chowdhury M.M., Nur-E-Elahi M., Chowdhury A.K., Faruque M.S., Wahiduzzaman M., Haque M.A. (2015). Pylorus Preserving Pancreaticoduodenectomy vs. Standard Whipple’s Procedure in Case of Carcinoma head of the Pancreas and Periampullary Carcinoma. Mymensingh Med. J..

[12] Yang C., Wu H.-S., Chen X.-L., Wang C.-Y., Gou S.-M., Xiao J., He Z.-Q., Chen Q.-J., Li Y.-F. (2014). Pylorus-Preserving Versus Pylorus-Resecting Pancreaticoduodenectomy for Periampullary and Pancreatic Carcinoma: A Meta-Analysis. Hoffmann A-C, ed. PLoS ONE.

[13] Hanna M.M., Gadde R., Allen C.J., Meizoso J.P., Sleeman D., Livingstone A.S., Merchant N., Yakoub D. (2016). Delayed gastric emptying after pancreaticoduodenectomy. J. Surg. Res..

[14] Kondo K., Chijiiwa K., Ohuchida J., Kai M., Fujii Y., Otani K., Hiyoshi M., Nagano M., Imamura N. (2013). Selection of prophylactic antibiotics according to the microorganisms isolated from surgical site infections (SSIs) in a previous series of surgeries reduces SSI incidence after pancreaticoduodenectomy. J. Hepatobiliary Pancreat. Sci..

[15] Kimura F., Shimizu H., Yoshidome H., Ohtsuka M., Kato A., Yoshitomi H., Nozawa S., Furukawa K., Mitsuhashi N., Sawada S. (2006). Increased Plasma Levels of IL-6 and IL-8 are Associated With Surgical Site Infection After Pancreaticoduodenectomy. Pancreas.

[16] Suragul W., Rungsakulkij N., Vassanasiri W., Tangtawee P., Muangkaew P., Mingphruedhi S., Aeesoa S. (2020). Predictors of surgical site infection after pancreaticoduodenectomy. BMC Gastroenterol..

[17] Fong Z.V., McMillan M.T., Marchegiani G., Sahora K., Malleo G., De Pastena M., Loehrer A.P., Lee G.C., Ferrone C.R., Chang D.C. (2016). Discordance Between Perioperative Antibiotic Prophylaxis and Wound Infection Cultures in Patients Undergoing Pancreaticoduodenectomy. JAMA Surg..

[18] Sugiura T., Mizuno T., Okamura Y., Ito T., Yamamoto Y., Kawamura I., Kurai H., Uesaka K. (2015). Impact of bacterial contamination of the abdominal cavity during pancreaticoduodenectomy on surgical-site infection. Br. J. Surg..

[19] Takahashi Y., Takesue Y., Fujiwara M., Tatsumi S., Ichiki K., Fujimoto J., Kimura T. (2018). Risk factors for surgical site infection after major hepatobiliary and pancreatic surgery. J. Infect. Chemother..

[20] Popp F.C., Popp M.C., Zhao Y., Betzler C., Kropf S., Garlipp B., Benckert C., Kalinski T., Lippert H., Bruns C.J. (2017). Protocol of the PANCALYZE trial: A multicenter, prospective study investigating the tumor biomarkers CXCR4, SMAD4, SOX9 and IFIT3 in patients with resected pancreatic adenocarcinoma to predict the pattern of recurrence of the disease. BMC Cancer.

[21] Su Z., Koga R., Saiura A., Natori T., Yamaguchi T., Yamamoto J. (2010). Factors influencing infectious complications after pancreatoduodenectomy. J. Hepatobiliary Pancreat. Sci..

[22] Hüttner F.J., Fitzmaurice C., Schwarzer G., Seiler C.M., Antes G., Büchler M.W., Diener M.K., Büchler M.W. (2014). Pylorus-preserving pancreaticoduodenectomy (pp Whipple) versus pancreaticoduodenectomy (classic Whipple) for surgical treatment of periampullary and pancreatic carcinoma. Cochrane Database of Systematic Reviews.

[23] Hackert T., Probst P., Knebel P., Doerr-Harim C., Bruckner T., Klaiber U., Werner J., Schneider L., Michalski C.W., Strobel O. (2018). Pylorus Resection Does Not Reduce Delayed Gastric Emptying After Partial Pancreatoduodenectomy. Ann. Surg..

[24] Horan T.C., Gaynes R.P., Martone W.J., Jarvis W.R., Emori T.G. (1992). CDC Definitions of Nosocomial Surgical Site Infections, 1992: A Modification of CDC Definitions of Surgical Wound Infections. Infect. Control Hosp. Epidemiol..

[25] Chen Q.J., He Z.Q., Yang Y., Zhang Y.S., Chen X.L., Yang H.J., Zhu S.K., Zhong P.Y., Yang C., Wu H.S. (2015). Is there comparable morbidity in pylorus-preserving and pylorus-resecting pancreaticoduodenectomy? A meta-analysis. J. Huazhong Univ. Sci. Technol. Med. Sci..

[26] Farnell M.B. (2009). Patient and hospital characteristics on the variance of perioperative outcomes for pancreatic resection in the United States—Invited critique. Arch. Surg..

[27] Alsfasser G., Leicht H., Günster C., Rau B.M., Schillinger G., Klar E. (2015). Volume–outcome relationship in pancreatic surgery. Br. J. Surg..

[28] Barreto S.G., Singh M.K., Sharma S., Chaudhary A. (2015). Determinants of Surgical Site Infections Following Pancreatoduodenectomy. World J. Surg..

[29] Zhang L., Liao Q., Zhang T., Dai M., Zhao Y. (2016). Blood Transfusion is an Independent Risk Factor for Postoperative Serious Infectious Complications After Pancreaticoduodenectomy. World J. Surg..

[30] Poruk K.E., Lin J.A., Cooper M.A., He J., Makary M.A., Hirose K., Cameron J.L., Pawlik T.M., Wolfgang C.L., Eckhauser F. (2016). A novel, validated risk score to predict surgical site infection after pancreaticoduodenectomy. HPB.

[31] Okano K., Hirao T., Unno M., Fujii T., Yoshitomi H., Suzuki S., Satoi S., Takahashi S., Kainuma O., Suzuki Y. (2015). Postoperative infectious complications after pancreatic resection. Br. J. Surg..

[32] Morikane K. (2017). Epidemiology and risk factors associated with surgical site infection after different types of hepatobiliary and pancreatic surgery. Surg. Today.

[33] De Pastena M., Paiella S., Marchegiani G., Malleo G., Ciprani D., Gasparini C., Secchettin E., Salvia R., Bassi C. (2017). Postoperative infections represent a major determinant of outcome after pancreaticoduodenectomy: Results from a high-volume center. Surgery.

[34] Sudo T., Murakami Y., Uemura K., Hashimoto Y., Kondo N., Nakagawa N., Ohge H., Sueda T. (2014). Perioperative Antibiotics Covering Bile Contamination Prevent Abdominal Infectious Complications after Pancreatoduodenectomy in Patients With Preoperative Biliary Drainage. World J. Surg..

[35] Gong L., Huang X., Wang L., Xiang C. (2020). The effect of preoperative biliary stents on outcomes after pancreaticoduodenectomy. Medicine.

[36] Chen Y., Ou G., Lian G., Luo H., Huang K., Huang Y. (2015). Effect of Preoperative Biliary Drainage on Complications Following Pancreatoduodenectomy. Medicine.

[37] Scheufele F., Schorn S., Demir I.E., Sargut M., Tieftrunk E., Calavrezos L., Jäger C., Friess H., Ceyhan G.O. (2017). Preoperative biliary stenting versus operation first in jaundiced patients due to malignant lesions in the pancreatic head: A meta-analysis of current literature. Surgery.

[38] Stauffer J.A., Onkendi E.O., Wallace M.B., Raimondo M., Woodward T.A., Lukens F.J., Asbun H.J. (2017). Standardization and streamlining of a pancreas surgery practice improves outcomes and resource utilization: A single institution’s 20-year experience. Am. J. Surg..

